# Individualised dosimetry for holmium-166 RE in patients with unresectable hepatocellular carcinoma; a multi-centre, interventional, non-randomised, non-comparative, open label, phase II study: RHEPaiR

**DOI:** 10.1136/bmjopen-2024-097066

**Published:** 2025-11-19

**Authors:** Maria Qurashi, Maria Martinez, Caroline Ward, Charlotte Wyard, Hooshang Izadi, Chloe Bowen, Sairah R Khan, Paul Tait, Maarten Smits, Joep de Bruijne, Robert Thomas, Marnix G E.H Lam, Rohini Sharma

**Affiliations:** 1 Imperial College London, London, UK; 2 Department of Surgery and Cancer, Hammersmith Hospital, London, UK; 3 Department of Mechanical Engineering and Mathematical Sciences, Oxford Brookes University, Oxford, Oxfordshire, UK; 4 Department of Nuclear Medicine, Hammersmith Hospital, London, UK; 5 Department of Interventional Radiology, Hammersmith Hospital, London, UK; 6 Department of Radiology and Nuclear Medicine, University Medical Center Utrecht, Utrecht, Netherlands; 7 Department of Hepatology, University Medical Center Utrecht, Utrecht, Netherlands; 8 University College London Hospitals NHS Foundation Trust, London, UK; 9 Nuclear medicine, Universitair Medisch Centrum Utrecht, Utrecht, Utrecht, Netherlands

**Keywords:** Safety, Quality of Life, Hepatobiliary tumours, Interventional radiology

## Abstract

**Introduction:**

Radioembolisation (RE) is gaining traction as a robust treatment option for patients with hepatocellular cancer (HCC) across all cancer stages. RE allows the delivery of targeted high-dose radiation directly to tumours, with relative sparing of the surrounding liver tissue. Traditionally, radiation has been delivered using ^90^Yttrium ([^90^Y]Y)-labelled microspheres, either glass or resin. The success of RE is dependent on the dose delivered to the tumour. When using [^90^Y]Y microspheres, dose prediction is calculated through a ^99m^Technitium ([^99m^Tc]Tc)-macroaggregated albumin (MAA) scan, which allows the calculation of the dose to be administered to the tumour. However, [^99m^Tc]Tc-MAA is not a true surrogate of [^90^Y]Y microspheres, and this will impact on the final dose delivered. [^166^Ho]Ho, like [^90^Y]Y, is a beta emitter but unlike [^90^Y]Y also emits gamma-radiation, allowing for quantitative nuclear imaging. The primary aim of this pilot study was to investigate the safety and efficacy of dosimetry-based individualised ^166^Holmium ([^166^Ho]Ho-RE) in patients with HCC.

**Methods and analysis:**

15 eligible participants will be recruited to receive [^166^Ho]Ho-RE. The primary objective is to establish the toxicity profile of dosimetry-based individualised [^166^Ho]Ho-RE. The secondary objective is to assess efficacy as measured by modified Response Evaluation Criteria in Solid Tumours (mRECIST) and Response Evaluation Criteria in Solid Tumours (RECIST) 1.1 criteria. Additional exploratory objectives include quality of life assessment and identification of a radiomic signature of response. The results from this study will be combined with the prospective iHEPAR study to form a larger analysis.

**Ethics and dissemination:**

The study has received approval from the East Midlands—Nottingham 1 Research Ethics Committee—approval number 23/EM/0239. The study will be performed in compliance with the Declaration of Helsinki and the principles of Good Clinical Practice. Signed informed consent will be obtained from each patient before study entry. The results will be disseminated through publication in a peer-reviewed scientific journal.

**Trial registration number:**

Clinicaltrials.gov NCT06302400.

**STRENGTHS AND LIMITATIONS OF THIS STUDY**

- Prospective study of a novel radioligand for radioembolisation (RE).
- Potential for more accurate delivery of RE to liver tumours.
- Small sample size.
- Single institution experience.

## Introduction

 Radioembolisation (RE) is gaining traction for the management of hepatocellular cancer (HCC) and is recommended by international and national bodies for the management of early and intermediate stage tumours.^1^ Glass or resin beads coated with [^90^Y]Y) or [^166^Ho]Ho-labelled synthetic beads are infused into the hepatic artery allowing the delivery of high-dose targeted radiotherapy directly to the tumour.

RE has been extensively studied in HCC as exemplified by the SARAH, SORAMIC and SIRveNIB studies.^2^,^4^ In both studies, patients were randomised to receive RE vs sorafenib, and in SORAMIC, RE was administered in combination with sorafenib. The studies were negative for their primary outcome of improved survival with RE. However, there are a number of key criticisms of the studies, the most critical being the lack of individualised dosimetry-based treatment planning. In both SORAMIC and SARAH studies, patients were prescribed activity based on body surface area (BSA).^5^ However, BSA dosing does not account for proposed treatment volume, absorbed dose and liver function, all of which can lead to potential under- or overdosing.^6 7^

The importance of individualised dosing was highlighted in the DOSISPHERE-01 trial^8^ that clearly demonstrated that individualised treatment planning with glass [^90^Y]Y -RE improved response rates and median overall survival compared with standard treatment planning in patients with HCC such that patients who received individualised doses had a median survival of 26.6 months (95% CI 11.7 to NR) compared with 10.7 months (95% CI 6 to 16.8) in those receiving dosing according to one compartment modelling. With regard to doses received, in the one compartment modelling group, the average dose to the perfused liver was 120±20 Gy, whereas the individualised dosimetry group received a tumorous dose of ≥205 Gy, while the normal liver received no more than 120 Gy. Importantly, no increase in toxicity was noted using an individualised approach. These results have been replicated by other retrospective analyses with the clear message that tumour doses should be maximised to improve clinical outcomes.^9^ Based on these studies, individualised dosimetry is now routinely used in the clinic with the aim of maximising dose delivered to the tumour with relative sparing of the surrounding liver tissue.

Prior to the therapeutic administration of radiolabelled beads, a small dose of a radiolabelled surrogate is injected during the work-up angiography and single-photon emission computed tomography (SPECT)/CT. The surrogate dose is used to determine the radiation dose to be delivered to both the tumour and surrounding normal liver (ie, dosimetry).^10^ As a pure beta-emitter, quantitative in vivo imaging of [^90^Y]Y -microspheres is challenging; instead, [^99m^Tc]Tc-MAA is used as a surrogate of [^90^Y]Y -microsphere distribution. [^166^Ho]Ho emits both beta-particles and gamma-radiation such that the [^166^Ho]Ho microspheres can themselves be used for dosimetry calculations during RE work-up. Moreover, due to the paramagnetic characteristics of [^166^Ho]Ho microspheres, they can be visualised on MRI allowing monitoring of the intra-hepatic behaviour of the microspheres.^11 12^

Elschot *et al*^13^ compared the performance of [^99m^Tc]Tc-MAA and [^166^Ho]Ho-scout pretreatment diagnostic imaging for the estimation of the lung shunt fraction in 14 patients. The study showed that [^99m^Tc]Tc-MAA planar imaging and [^99m^Tc]Tc-MAA SPECT/CT imaging significantly overestimate lung absorbed doses, whereas [^166^Ho]Ho-scout SPECT-CT imaging accurately predicts absorbed doses. These findings have recently been confirmed in a further study of 37 patients.^14^ In another study, the agreement between the intrahepatic distribution of [^166^Ho]Ho scout and [^166^Ho]Ho therapeutic dose was compared with the distribution of [^99m^Tc]Tc-MAA and [^166^Ho]Ho-therapeutic dose, illustrating that [^166^Ho]Ho scout had a superior predictive value over [^99m^Tc]Tc-MAA.^15^

The HEPAR primary study established the safety and efficacy of [^166^Ho]Ho -RE in patients with HCC.^16^ In this study, 31 patients were dosed using a one compartment model where the average dose delivered to the perfused liver was 60 Gy. 19 serious adverse events occurred, of which four events in three patients were related to treatment (three possibly related and one definitely related). Per protocol, individual dosimetry was not employed. We therefore aim to explore the safety and toxicity profile of dosimetry-based individualised [^166^Ho]Ho-RE in patients with HCC with the aim of optimising tumour dose to >150 Gy while keeping normal liver doses to 45–60 Gy or under. The results of this study will be combined with the identical iHEPAR study (NCT05114148), a single-centre study, the primary aim of which is to ascertain the toxicity profile of individualised [^166^Ho]Ho-RE in patients with HCC.

## Methods and analysis

### Trial objectives

The primary objective is to determine the incidence of adverse and serious adverse device events (ADEs/SADEs) overall and by severity, graded by the National Cancer Institute—Common Toxicity Criteria (NCI-CTC v5.0). The co-primary objective is to determine the objective response rate (ORR) with [^166^Ho]Ho-RE, defined as the rate of best overall response (which includes complete responses and partial responses) in the treated liver volume as determined by RECIST 1.1 and mRECIST. In addition, the total hepatic response and overall response will be assessed according to RECIST 1.1 and mRECIST.

The secondary objectives are quality of life (QoL) as determined using the European Organisation for Research and Treatment of Cancer (EORTC) questionnaires QLQ-C30 and HCC18 at baseline and every 3 months. Exploratory endpoints include the assessment of radiomics in the prediction of treatment response determined by CT scans of the liver at baseline, 12 weeks post RE and at disease progression. Biodistribution and dosimetry received to tumour and normal liver will be reported. The results of this study will be combined with the identical iHEPAR study.

### Eligibility criteria

Participants will be recruited from Hammersmith Hospital, Imperial College Healthcare NHS Trust. Patients will be provided with a verbal and written explanation of the trial and given the opportunity to discuss all aspects of the trial with both the clinician and research nurse. Patients who provide written consent to participate in the trial after at least 24 hours of consideration will be registered and therefore eligible to proceed to further assessment.

To be eligible, patients must have a radiological or histological diagnosis of HCC not amendable to any curative options including transplant, surgery or radiofrequency ablation. Patients with up to Child Pugh B7 liver function and liver dominant disease will be enrolled. Detailed inclusion and exclusion criteria are outlined in online supplemental table 1.

### Study procedures

Patients will undergo baseline tumour imaging including chest, abdominal and pelvic CT scan and MRI scan of the liver at screening (table 1). Up to 4 weeks following consent, patients will undergo angiography of the hepatic artery to assess the presence of aberrant vessels arising from hepatic arteries. If present, protective coiling of any extrahepatic branch (eg, aberrant segment IV and right gastric artery) will be undertaken. In the same session, a scout dose of maximum 250 MBq ^166^Ho-labelled microspheres, QuiremScout (Terumo Europe), will be injected into the hepatic artery via the same catheter position chosen for the scheduled RE session in order to calculate the hepatopulmonary shunt fraction. In cases of selective treatments, less activity will be injected for the scout, proportionally to the volume treated. Tracer distribution will be evaluated using [^166^Ho]Ho SPECT imaging. Following completion of the angiogram and scout imaging, a decision will be made regarding the dose to be delivered. The individualised treatment plan will attempt to optimise doses to achieve at least a tumour dose of 150 Gy while keeping normal liver doses to 45–60 Gy or lower. QuiremSpheres (Terumo Europe) will be administered up to 2 weeks following these investigations.

**Table 1:** Treatment schedule

Procedures	Screening		Treatment	follow-up			
	Screening	Scout		W3	W6	M3	M6
Informed consent	X						
Inclusion/exclusion criteria	X	X	X				
Demographics	X						
Physical exam and clinical performance status (ECOG)	X					X	X
EORTC QLQ C30+HCC18	X					X	X
CT scan^*^	X						
MRI scan^†^	X		X			X	X
Tumour assessments (mRECIST)	X					X	X
SPECT/CT		X	X^‡^				
Angiography		X	X				
Scout dose		X					
Therapeutic dose			X				
Blood tests	X	X	X	X^§^	X^§^	X^§^	X^§^
(S)AE’s+Con Med	X			X	X	X	X

* CT scan chest + pelvis

† MRI liver with hepatic biliary contrast

‡ 3-4 days after treatment

§ No coagulation blood tests required

ECOG, Eastern Cooperative Oncology Group; EORTC, European Organisation for Research and Treatment of Cancer; mRECIST, modified Response Evaluation Criteria in Solid Tumors; SPECT, single-photon emission computed tomography.

Patients will be admitted the day of the procedure and discharged the same day. In patients with bilobar disease, the first treatment will be administered to the lobe with the greatest tumour burden. Treatment of the contralateral lobe will be scheduled for 4–6 weeks after the first treatment. Following administration of QuiremSpheres, patients will receive standard of care post-RE treatment to prevent radiation-induced hepatitis and gastric complications: reducing dose of dexamethasone 8 mg daily for 1 week, 6 mg daily for the following week, 4 mg daily the week after that and 2 mg daily for 1 final week, proton pump inhibitor 60 mg daily and ursodeoxycholic acid 250 mg every night. A post-therapy SPECT will be performed 3–4 days after QuiremSpheres administration.

Patients will be reviewed in week 3, week 6 and on a 3 monthly basis for 6 months or until disease progression, withdrawal from the trial or death (whichever comes first). Blood tests will be performed at each visit for safety. Tumour imaging will be repeated 3-monthly post-RE for 6 months. The same method for assessment at baseline must then be used at all subsequent time points. mRECIST criteria will be used to determine patient response to treatment and ORR. QoL questionnaires (EORTC QLQ-C30) and EORTC HCC18 will be completed at baseline, and 3-monthly post-RE until trial completion.

### Safety

All adverse events (AEs) and adverse reactions (ARs) will be reported in a timely fashion. AEs and ARs, whether expected or not, will be collected and recorded during clinical assessments at weeks 3 and 6 and 3-monthly for 6 months (NCI-CTC v5.0). When serious adverse events (SAEs) occur, an SAE form will be completed within 24 hours. The chief investigator will determine whether SAEs were ‘related’ (resulting from the administration of the study treatment or procedure) or ‘unexpected’ (an event not listed in the study protocol as an anticipated occurrence) and report this to the research ethics committee. Any questions concerning AE reporting will be directed to the chief investigator in the first instance. The chief investigator will notify the sponsor of all SAEs that occur.

### Data collection

Data will be collected using trial-specific patient report forms. As far as possible, missing data will be chased. For the primary analysis, there will be no data imputation for missing data in the primary endpoint. All patient-related data will be held in a secure password-protected electronic database that will only be accessible by the research team.

### Translational endpoints

Using established methodologies,^17^ we will extract radiomic features and correlate these with clinical outcome measures using lasso regression. Images from RHEPaiR will be combined with iHEPAR for analysis. This will allow us to prospectively identify radiologic predictors of outcome to RE.

### Statistical analysis

As this is a pilot study, no formal sample size calculation will be undertaken, and 15 patients will be recruited. Patients enrolled will be combined with the prospective iHEPAR study, which seeks to enrol 30 patients. iHEPAR has an 85% power to detect unacceptable toxicity occurring in 25% of patients. Participants failing screening will not count towards recruitment figures.

A full statistical analysis plan will be drawn up prior to database lock and any data interpretation. No interim analysis is scheduled to occur. The final analysis will be conducted at the end of the study once recruitment and treatment are complete.

The efficacy analysis will consist of the full analysis set (FAS), ie, all patients who received RE. The safety analysis will also be conducted in the FAS. The frequency of ADEs and SADEs will be assessed for severity (NCI-CTCAE v5.0), expectedness, seriousness and causal relationship to treatment. ADEs will be summarised by toxicity, type and timing. An estimation of ORR and its 95% CI will be reported. If appropriate and required, a per-protocol analysis set will be defined/finalised/approved prior to the final analysis on which the primary analysis will be repeated.

Histograms and box plots will be used to check the distribution and possible outliers for continuous variables. Continuous variables that follow a normal distribution will be summarised using means and SD. Skewed continuous variables will be summarised using medians and interquartile ranges. Categorical variables will be summarised using frequencies and percentages.

Any deviation(s) from the final statistical plan in the final analysis will be described and justification given in the final report.

### Patient and public involvement

Patients were not involved in the development of the protocol.

## Ethics and dissemination

The study was reviewed and approved by the East Midlands—Nottingham 1 Research Ethics Committee (23/EM/0239)(Nottingham1.rec@hra.nhs.uk), ARSAC committee (ARSAC ID AA-6621) and the UK Health Research Authority (IRAS ID 326861). The study is performed in compliance with the Declaration of Helsinki and the Principles of Good Clinical Practice. Signed informed consent is obtained from each patient before study entry (online supplemental material). Results from this study will be published in peer-review journals. Data generated can be made available from the authors following the approval of a data sharing agreement.

## Discussion

Personalised dosimetry is central to achieving clinical benefit in patients with HCC.^18^ Therapeutic delivery of [^90^Y]Y-labelled microspheres is routinely used in clinical practice, with [^99m^Tc]Tc-MAA used as a surrogate of dose distribution. The use of a surrogate has been shown to be inaccurate with regard to calculating the dose delivered to the tumour.^2 3^ As both a beta and gamma-emitter, [^166^Ho]Ho-labelled microspheres can be used for both dosimetry and for therapy, improving the accuracy of dose delivered. However, individualised treatment planning inherently leads to treatment doses that deviate from the currently approved ‘one-size-fits-all’ approach (ie, 60 Gy average absorbed dose in the perfused liver volume for all patients.^16^ Therefore, the safety of individualised ^166^Ho-RE needs to be evaluated especially in this patient group, the majority of whom have a background of chronic liver disease.

The results of this work will be combined with the identical iHEPAR study being conducted in the Netherlands, increasing the power of both studies and allowing validation of the technology across countries. Combined final analysis will allow determination of radiation safety thresholds. These thresholds will be used in subsequent randomised controlled studies of [^166^Ho]Ho-RE for HCC. The approach of using a harmonised protocol across countries reduces the costs of a multisite study while still maintaining the rigour needed to combine results across sites. Moreover, it allows sites in different countries to gain experience in a novel technology. RHEPaiR will also generate much needed local knowledge for regulatory approval within the UK. This trial will add prospective clinical outcomes to a space dominated by retrospective work particularly with regard to dosimetry, giving further support for the use of RE in HCC. Moreover, imaging biomarker work for the prediction of clinical outcome in particular radiomics will be studied, which may aid in treatment choice for patients moving forward.

## Supplementary material

10.1136/bmjopen-2024-097066 online supplemental file 1
