# Supplementary material for: Individualised dosimetry for holmium-166 RE in patients with unresectable hepatocellular carcinoma; a multi-centre, interventional, non-randomised, non-comparative, open label, phase II study: RHEPaiR
Source: BMJ Open. 2025 Nov 19;15(11):e097066. doi: 10.1136/bmjopen-2024-097066 (PMC12636873; doi:10.1136/bmjopen-2024-097066)
Supplement: online supplemental file 1 [file bmjopen-15-11-s001.docx]

**Supplementary Table 1: Inclusion and exclusion criteria**

| **Inclusion Criteria** | **Exclusion Criteria** |
| --- | --- |
| In order to be eligible to participate in this study, a subject must meet all of the following criteria:   1. Patients must have given written informed consent. 2. Female or male aged 18 years and over. 3. Diagnosis of HCC established according to AASLD criteria: nodule >1 cm in a patient at risk for HCC, with combination of arterial hypervascularity and venous or delayed phase wash-out on multiphase CT-scan or MRI-scan. LR-5 and LR- 4 based on Liver Imaging Reporting and Data System can be included. 4. No curative treatment options (resection, transplant, or in case of solitary tumour, RFA). 5. Life expectancy of at least 6 months. 6. ECOG Performance status 0-1 7. Liver-dominant disease (maximum 5 lung nodules all ≤1.0 cm, solitary clinically stable adrenal metastasis, and mesenteric or portal lymph nodes all ≤2.0 cm are accepted). 8. Child-Pugh class A5-6 or B7. 9. At least one measurable liver lesion according to the modified RECIST criteria. ([26](#_bookmark24)) 10. Negative pregnancy test for women of childbearing potential. Female patients of childbearing potential should use a highly effective acceptable method of contraception (oral contraceptives, barrier methods, approved contraceptive implant, long-term injectable contraception, intrauterine device or tubal ligation) or should be more than 1 year postmenopausal or surgically sterile during their participation in this study (from the time they sign the consent form), to prevent pregnancy. | A potential subject who meets any of the following criteria will be excluded from participation in this study:   1. Evidence of significant extrahepatic disease (MRI-scan liver and multiphase abdominal CT as well as a thoracic CT are routinely performed at screening) not including minimal disease as defined as maximum 5 lung nodules all ≤1.0 cm, solitary clinically stable adrenal metastasis, and mesenteric or portal lymph nodes all ≤2.0 cm are accepted. 2. Hepatic radiation therapy within the last 4 weeks before the start of study therapy. 3. Previous or current treatment with RE. Previous treatment with TACE, surgery, RFA, and previous or current treatment with systemic treatment are allowed. 4. Major surgery within 4 weeks or incompletely healed surgical incision before starting study therapy. 5. Serum bilirubin > 34 umol/L in the absence of a reversible cause 6. Glomerular filtration rate <35 ml/min. 7. Non-correctable INR >1.5 in case of femoral approach (as opposed to radial). 8. Platelet count <50 109/l. 9. Significant cardiac event (e.g., myocardial infarction, superior vena cava (SVC) syndrome, New York Heart Association (NYHA) classification of heart disease ≥2) within 3 months before entry, or presence of cardiac disease that in the opinion of the investigator increases the risk of ventricular arrhythmia. 10. Pregnancy or breastfeeding. 11. Patients suffering from psychic disorders that make a comprehensive judgment impossible, such as psychosis, hallucinations and/or depression. 12. Patients who are declared incapacitated. 13. Previous enrolment in the present study. 14. Male patients who are not surgically sterile or do not use an acceptable method of contraception during their participation in this study (from the time they sign the consent form), to prevent pregnancy in a partner. 15. Evidence of untreated, clinically significant grade 3 portal hypertension (i.e. large varices at oesophagi-gastro-duodenoscopy). In these cases, therapy with non-selective beta-blocker (propranolol) or rubber band ligation should be instituted according to accepted guidelines. In case of small varices, prophylactic propranolol is advised. 16. Portal vein thrombosis (tumour and/or bland) of the main branch (diagnosed on contrast enhanced transaxial images). Involvement of the right or left portal vein branches and more distal is accepted. 17. Untreated active hepatitis. For Hepatitis B this is defined by positive HBsAg or HBV DNA and for Hepatitis C as positive anti-HCV antibodies and HCV RNA. In case of detectable viral HBV load, appropriate treatment should be instituted. 18. Transjugular intrahepatic portosystemic shunt (TIPS). 19. Body weight over 150 kg (because of maximum table load). 20. Severe allergy for intravenous contrast used (Visipaque®)(because of CT evaluation, pre- treatment angiography and treatment angiography). 21. Lung shunt >30 Gy, as calculated using scout dose SPECT/CT. 22. Extrahepatic deposition of scout dose activity. Activity in the falciform ligament, portal lymph nodes and gallbladder is accepted. |
