# Supplementary material for: Individualised dosimetry for holmium-166 RE in patients with unresectable hepatocellular carcinoma; a multi-centre, interventional, non-randomised, non-comparative, open label, phase II study: RHEPaiR
Source: BMJ Open. 2025 Nov 19;15(11):e097066. doi: 10.1136/bmjopen-2024-097066 (PMC12636873; doi:10.1136/bmjopen-2024-097066)
Supplement: Supplementary file 2 [file bmjopen-2024-097066.reviewer_comments.pdf]

PEER REVIEW HISTORY

BMJ Open publishes all reviews undertaken for accepted manuscripts. Reviewers are asked to complete a checklist review form and are provided with free text boxes to elaborate on their assessment. These free text comments are reproduced below.

ARTICLE DETAILS

Title (Provisional)

Individualized dosimetry for holmium-166 RE in patients with unresectable hepatocellular carcinoma; a multi-centre, interventional, non-randomized, non-comparative, open label, phase II study: RHEPaiR

Authors

Qurashi, Maria; Martinez, Maria; Ward, Caroline; Wyard, Charlotte; Izadi, Hooshang; Bowen, Chloe; Khan, Sairah R; Tait, Paul; Smits, Maarten; de Bruijne, Joep; Thomas, Robert; Lam, Marnix G.E.H.; Sharma, Rohini

---

VERSION 1 - REVIEW

**Reviewer**	**1**
**Name**	**Tateishi, Ryosuke**
**Affiliation**	**The University of Tokyo, Department of Gastroenterology**
**Date**	**26-Jun-2025**
**COI**	

This is a well-written and well-structured study protocol describing the design of the RHEPaiR trial, a phase II, non-randomized, non-comparative, open-label, multi-center study evaluating the safety and efficacy of dosimetry-based individualized holmium-166 radioembolisation (166Ho-RE) in patients with unresectable hepatocellular carcinoma (HCC). The rationale for the study is sound, with clear justification provided based on recent trials and limitations of current standard dosimetry practices.

The manuscript meets the core principles of the SPIRIT 2013 guideline for protocol publications. However, several issues should be addressed.

Major concern

Ethical issues

1. While the protocol states that ethical approval has been obtained, it would benefit from clarification on the justification for including patients with extrahepatic metastases in a non-systemic treatment trial. This raises potential ethical concerns, particularly regarding the

balance of risks and benefits, and whether patients are adequately informed about standard care alternatives.

Inclusion and exclusion criteria

2. The inclusion and exclusion criteria regarding extrahepatic disease should be harmonized for clarity and consistency (see Table 1, items 7 and 1).

3. The definition of untreated active hepatitis should be clarified. Please also state the definition regarding hepatitis C.

Data Monitoring Committee (SPIRIT 21a)

4. The manuscript does not state whether a Data Monitoring Committee (DMC) has been established. Please clarify whether a DMC is established. If not, provide a rationale for its omission.

Confidentiality and Data Access (SPIRIT 27, 29)

5. There is no statement on how participant data will be secured or who will have access to the final dataset. Please include a section on data protection measures and access restrictions in the final dataset.

Ancillary and Post-Trial Care (SPIRIT 30)

6. There is no mention of post-trial care or compensation for trial-related injury. Please clarify whether any provisions exist for post-trial care or compensation in case of harm.

Concurrent iHEPAR trial

7. Given the close relationship between this trial and the concurrent iHEPAR trial, it would be helpful to include a brief description of the iHEPAR study design along with its ClinicalTrials.gov identifier (NCT05114148).

Minor comments

8. Please consider spelling out the abbreviation "RE" (radioembolisation) at its first mention in the abstract.

9. In the statistical analysis section, the word “with” should be removed.

VERSION 1 - AUTHOR RESPONSE

Reviewer: 1
Dr. Ryosuke Tateishi, The University of Tokyo
Comments to the Author:
This is a well-written and well-structured study protocol describing the design of the RHEPaiR trial, a phase II, non-randomized, non-comparative, open-label, multi-center study evaluating the safety and efficacy of dosimetry-based individualized holmium-166 radioembolisation (166Ho-RE) in patients with unresectable hepatocellular carcinoma

(HCC). The rationale for the study is sound, with clear justification provided based on recent trials and limitations of current standard dosimetry practices.
The manuscript meets the core principles of the SPIRIT 2013 guideline for protocol publications. However, several issues should be addressed.

Major concern
Ethical issues
1. While the protocol states that ethical approval has been obtained, it would benefit from clarification on the justification for including patients with extrahepatic metastases in a non-systemic treatment trial. This raises potential ethical concerns, particularly regarding the balance of risks and benefits, and whether patients are adequately informed about standard care alternatives.
Many thanks for raising this issue. As stated in the protocol and consistent with other published studies in HCC namely SARAH, SORAMIC, SIRveNIB, we included patients with minimal extra-hepatic disease as stated in the protocol; the rationale being that the prognosis of these patients is dictated by the hepatic disease burden rather than minimal metastatic disease, and where there may be doubt as to the nature of the lesions particularly a small node. This is consistent with EASL guidelines whereby SIRT is an option for patients with minimal extrahepatic disease. We do agree that if a patient has definitive extrahepatic disease they would not be eligible for the trial and would be offered systemic therapy and this is clearly indicated in the protocol.

Inclusion and exclusion criteria
2. The inclusion and exclusion criteria regarding extrahepatic disease should be harmonized for clarity and consistency (see Table 1, items 7 and 1).
This has been addressed.

3. The definition of untreated active hepatitis should be clarified. Please also state the definition regarding hepatitis C.
This has been added to Table 1.

Data Monitoring Committee (SPIRIT 21a)
4. The manuscript does not state whether a Data Monitoring Committee (DMC) has been established. Please clarify whether a DMC is established. If not, provide a rationale for its omission.
As this is a pilot study of a device that is registered for routine use albeit in different indications the regulatory authorities ie MHRA did not mandate the use of a DMC.

Confidentiality and Data Access (SPIRIT 27, 29)
5. There is no statement on how participant data will be secured or who will have access to the final dataset. Please include a section on data protection measures and access restrictions in the final dataset.
This has now been addressed and a statement added to page 5 "All patient related data will be held in a secure password protected electronic database that will only be accessible by the research team."

Ancillary and Post-Trial Care (SPIRIT 30)
6. There is no mention of post-trial care or compensation for trial-related injury. Please clarify whether any provisions exist for post-trial care or compensation in case of harm.
As per standard Imperial College procedures, any injury or illness pertaining to trial conduct

will be compensated as per the patient information sheet.

Concurrent iHEPAR trial
7. Given the close relationship between this trial and the concurrent iHEPAR trial, it would be helpful to include a brief description of the iHEPAR study design along with its ClinicalTrials.gov identifier (NCT05114148).

This has been addressed paragraph 1, page 4 “The results of this study will be combined with the identical, iHEPAR study (NCT05114148) a single centre study, the primary aim of which is to ascertain toxicity profile of individualized [166Ho]Ho-RE in patients with HCC.”

Minor comments
8. Please consider spelling out the abbreviation "RE" (radioembolisation) at its first mention in the abstract.
This has been addressed.

9. In the statistical analysis section, the word “with” should be removed.

This has been addressed.

VERSION 2 - REVIEW

**Reviewer**	**1**
**Name**	**Tateishi, Ryosuke**
**Affiliation**	**The University of Tokyo, Department of Gastroenterology**
**Date**	**27-Aug-2025**
**COI**	

The authors revised the manuscript appropriately according to the reviewer's comments, except for the following point.

Supplementary Table 1, exclusion criteria, item 17: "positive anti-HCV and HCV RNA" is correct because anti-HCV remains positive after sustained virological response after anti-HCV treatment.

VERSION 2 - AUTHOR RESPONSE

Reviewer: 1
Dr. Ryosuke Tateishi, The University of Tokyo
Comments to the Author:
The authors revised the manuscript appropriately according to the reviewer's comments, except for the following point.

Many thanks

Supplementary Table 1, exclusion criteria, item 17: "positive anti-HCV and HCV RNA" is correct because anti-HCV remains positive after sustained virological response after anti-HCV treatment.

This has been corrected
